# Pathological features and clinical behavior of Lynch syndrome-associated ovarian cancer

**DOI:** 10.1016/j.ygyno.2017.01.005

**Published:** 2017-03

**Authors:** N.A.J. Ryan, D.G. Evans, K. Green, E.J. Crosbie

**Affiliations:** a Division of Molecular and Clinical Cancer Sciences, Faculty of Biology, Medicine and Health, University of Manchester, St Mary's Hospital, Manchester, UK; b Division of Evolution and Genomic Medicine, University of Manchester, St Mary's Hospital, Manchester, UK; c Manchester Centre for Genomic Medicine, Central Manchester University Hospitals NHS Foundation Trust, Manchester Academic Health Science Centre, Manchester, UK; d Department of Obstetrics and Gynaecology, Central Manchester University Hospitals NHS Foundation Trust, Manchester Academic Health Science Centre, Manchester, UK

## Abstract

**Objective:**

Lynch syndrome (LS) is an inherited tumor predisposition condition caused by mutations in the mismatch repair (MMR) genes. Mutation carriers are at increased risk of various malignancies, including ovarian cancer (OC). Relatively little is known about the pathological features and clinical behavior of LS associated OC.

**Methods:**

We analyzed the data of 1047 proven MMR mutated individuals from a prospectively maintained database at a large referral center for genomic medicine in the North West of England. Data were crosschecked with pathology reports, the National Cancer Registry and death certificates, where appropriate. Data from gynecological surveillance and risk reducing surgery were analyzed.

**Results:**

We identified 53 cases of LSAOC in proven MMR mutated individuals. The cumulative risk of LSAOC was 20% at age 80 in those who retained their ovaries. LSAOC presented at an earlier age (average 51, range 24–70 years) than sporadic OC. The predominant histological subtype was endometrioid adenocarcinoma (53%). Most cases presented early (85% at stage I/II vs. 15% at stage III/IV, *p* < 0.001) and overall survival was excellent (80% 5-year survival), however, patients with advanced disease had a poor prognosis (40% 5-year survival). Most women were found to have LS after their OC diagnosis, however, two were detected at Stage 1c through gynecological surveillance and a further three were detected following surgery for screen-detected synchronous endometrial pathology.

**Conclusion:**

The predominance of early stage disease in LSAOC is linked to its good prognosis. We support risk-reducing surgery for women whose families are complete especially if undertaking hysterectomy for endometrial risk, and ovarian surveillance as part of gynecological screening for those who have not.

## Highlights

• Lynch syndrome-associated ovarian cancer (LSAOC) is rare and difficult to study.
• This is the largest reported series of OC from proven Lynch syndrome carriers.
• Endometrioid OC was most common, followed by high grade serous, clear cell and mixed histology.
• Most LSAOC was detected at stage 1 and overall 5-year survival was excellent at 80%.
• Surveillance found 2 LSAOC; 3 more were diagnosed following surgery for screen-detected endometrial cancer.

## 1 Introduction

Lynch syndrome (LS) is an autosomal dominant tumor predisposition condition caused by mutations in the mismatch repair (MMR) genes. The syndrome was first described in 1913 by Warthin and further delineated by Lynch in 1966 [1]. Lynch syndrome is thought to affect 1:2000 to 1:370 individuals [2], [3]. As a result of a dysfunctional MMR system, cells become hyper-mutated with a high microsatellite (MSI-H) instability phenotype; mutations in the oncoprotective genes eventually lead to neoplastic changes and tumorigenesis. Mutation carriers are at high risk of colorectal cancer (CRC), endometrial cancer (EC) and a spectrum of other malignancies, including ovarian cancer (OC). Diagnosis is suspected from clinical presentation and family history, and confirmed with immunohistochemistry, MSI analysis and ultimately genomic sequencing of the known MMR genes.

OC is the leading cause of death from gynecological cancer in the developed world [4]. Symptoms are often vague at onset leading to a delayed diagnosis. The lifetime risk of Lynch syndrome-associated OC (LSAOC) is in the region of 6–14% [4], [5], with around 2% of all ovarian cancers due to Lynch syndrome, although studies are lacking and sample sizes are small [6]. There is a clinical imperative to identify LSAOC since surveillance strategies can be used to identify and treat premalignant and early stage cancers of other anatomical sites, particularly those of the rectum and colon. In addition, potentially affected relatives can be offered diagnostic testing. Lynch syndrome-associated colorectal cancers have distinctive pathological features that arouse diagnostic suspicion [7], [8], [9], however, the clinicopathological features of LSAOC remain poorly defined. Here we present the largest case series of LSAOC in known Lynch syndrome mutation carriers. We explore the clinicopathological features of these tumors, associations between the genetics and the disease and disease-specific survival analysis.

## 2 Methods

**Fig. 1:**
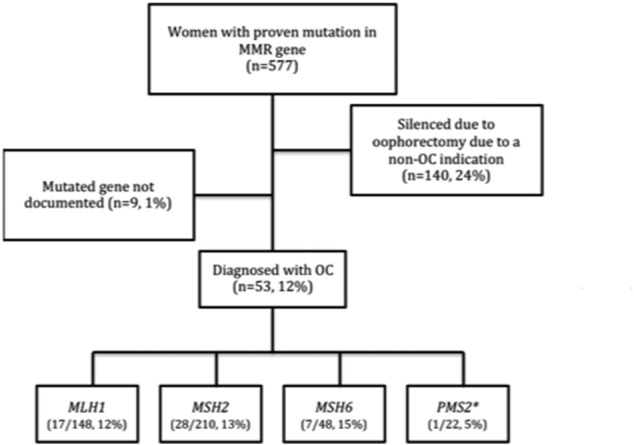
A flow diagram outlining the inclusion stratification for the study. Asterix (*) denotes patient with homozygous *PMS2* mutation, excluded from further analysis.

The clinical records of the Manchester Centre for Genomic Medicine, a large tertiary referral genetics center in the North West of England in the United Kingdom, were searched for cases of LSAOC. This was facilitated by an electronic prospectively maintained clinical database, which is maintained by a dedicated data manager. All those included on the database have given formal consent to have their data analyzed anonymously and published. The genetic center serves a population of 5.6 million people. In total 1047 proven mutation carriers are included in the database. Of these, 577 are women. Only those with a confirmed diagnosis of Lynch syndrome based on germline sequencing were included in this study. Fig. 1 outlines the numbers of patients included and excluded at each stage of stratification. Patients at potential risk of LS are referred as either affected or unaffected individuals where there is young onset of colorectal, endometrial and/or ovarian cancer or a pattern of these cancers suggestive of LS. Testing is usually directed by immunohistochemistry (IHC) of relevant tumors with initial testing by next generation sequencing of *MSH2*, *MLH1* and *MSH6*. *PMS2* is only tested if there is loss of PMS2 protein on IHC. All mutations are assessed for pathogenicity utilizing the INSiGHT dataset. All Lynch syndrome diagnostic tests are performed within a nationally accredited genetics laboratory and with full consent from patients.

Women with known Lynch syndrome and those considered at risk of LS or an LS-like syndrome are offered gynecological cancer surveillance even in the absence of a proven MMR mutation. This has been routine practice since 1997. Each year, women undergo an outpatient hysteroscopy and transvaginal ultrasound surveillance for endometrial pathology. Ovarian surveillance by ultrasound and serum cancer antigen 125 (CA125) testing is individualized according to family history. There are currently 87 women enrolled in this surveillance program, approximately two thirds of whom have not yet had their Lynch syndrome status tested or confirmed.

We collated clinical data from the database and the patients' case notes for all women with proven LSAOC. Pedigree data were used to assess whether they met the Bethesda guideline criteria. Patients were tracked from the time of LSAOC diagnosis and censored at the time of death or last follow up. Individual date of death data were collected from the National Cancer Registry during the course of the patient's clinical care. In addition, cause of death was established from official death certificates, where appropriate. Pathology reports were collected and cancers were staged, on the basis of these original reports, in line with the International Federation of Gynecology and Obstetrics (FIGO) 2009 criteria. This allowed for standardization of the disease picture. All data were anonymized the time of data extraction.

Descriptive statistics were generated, including means, confidence intervals and proportions. Statistical analysis was performed using a combination of both Graphpad Prism version 7 (California USA) and StataSE version 13 (StataCorp Texas USA) software. Statistical hypothesis testing was completed with the use of analysis of variance or student *t*-test, as appropriate. Results were tabulated or presented graphically. Percentage survival was generated with the use of Kaplan-Meier algorithm.

## 3 Results

**Fig. 2:**
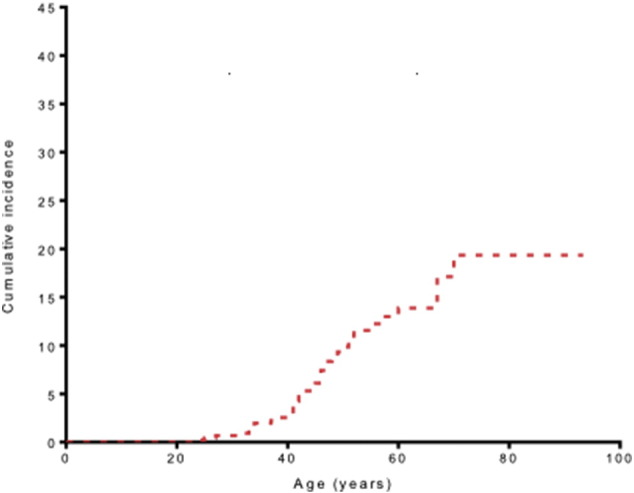
Lifetime cumulative incidence of ovarian cancer in our cohort (*n* = 577). Those who had undergone bilateral oophorectomy (*n* = 140) were censored at the date of oophorectomy.

In our cohort, the lifetime cumulative risk of LSAOC was 2% at 40 years of age, 15% at 60 years and 20% at 80 years of age (Fig. 2). Twenty-four percent of women were censored from analysis at the time of bilateral salpingo-oophorectomy for LSAEC prevention or treatment. In total, 53 LSAOC tumors were identified. The mean age of diagnosis was 51 years (range 24–70 years). Diagnosis of OC dated from 1956 to 2015. Mean period of follow up, from the time of LSAOC diagnosis, was 64 months. Three of the four Lynch syndrome genes were represented in the population with the exception of *PMS2*. One individual carried a bi-allelic mutation in *PMS2* giving her a diagnosis of constitutional mismatch repair deficiency (CMMRD) rather than Lynch syndrome, and was excluded from analysis. In total, there were 17 *MLH1*, 28 *MSH2* and 7 *MSH6* proven mutation carriers. The mean age of LSAOC in *MLH1* was 48 years, in *MSH2* it was 52 years and in *MSH6* the average was 53 years. There was no significant difference between age at diagnosis of LSAOC and mutated gene (*p* = 0.51 ANOVA), although numbers are small. Of the 36 women with complete datasets (Table 1), eight met the Bethesda criteria for diagnosis of Lynch syndrome; this constitutes just 22% of the cohort.

The histopathological features of the LSAOC tumors are presented in Table 2. There was a preponderance of high-grade endometrioid tumors (*n* = 19), with them constituting 53%. This was followed high-grade serous adenocarcinomas (*n* = 6), and mixed tumors (*n* = 4) constituting 17% and 11% respectively. Clear cell carcinoma (*n* = 4) constituted 11%. There were singular recorded cases of anaplastic neuroendocrine (*n* = 1), yolk sac (*n* = 1) and carcinosarcoma (*n* = 1) tumors, each constituting 3%. The stage of the disease was verified in 35 cases. Most patients presented with stage 1 disease [stage 1a, 12 (34%); stage 1b, 4 (11%); stage 1c, 7 (20%)]. Seven women (20%) presented with stage 2 disease, 4 (11%) with stage 3 and 1 (3%) with stage 4 disease, respectively.

**Fig. 3:**
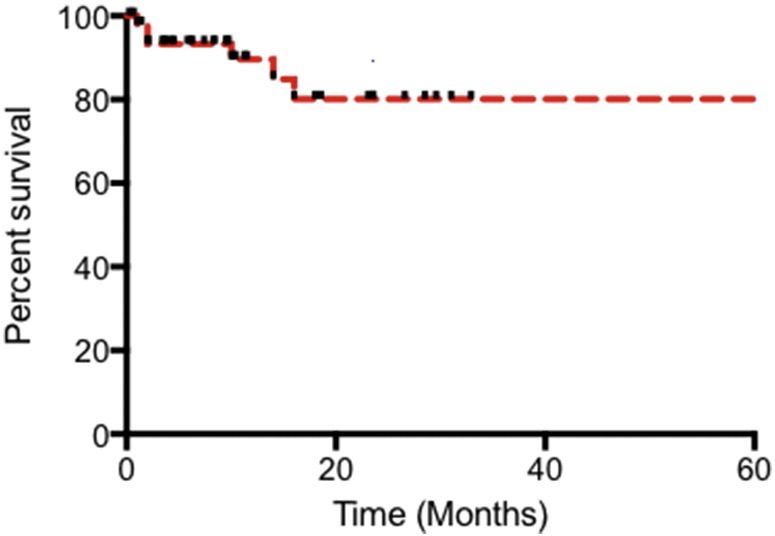
Overall survival for women diagnosed with Lynch syndrome associated ovarian cancer in our cohort.

**Fig. 4:**
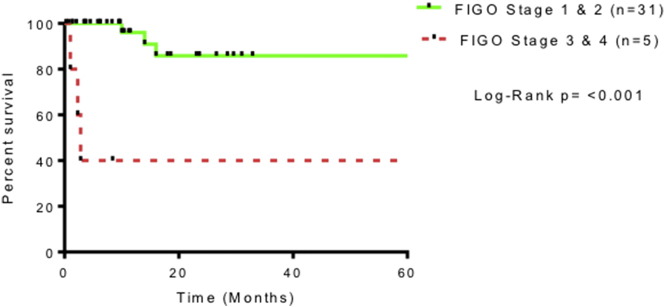
Survival in LSAOC stratified by FIGO 2009 stage.

Synchronous endometrial cancer or atypical hyperplasia was seen in 9 women (25%). Contemporaneous histological opinion favored synchronous primary ovarian pathology rather than metastasis. There were 11 deaths (29%) recorded within the cohort. Of these, six (17%) had OC documented as the primary cause of death. Three of the six were stage 1, and the remaining three were documented as stage 2, 4 and unknown, respectively. None of these cases underwent a formal post-mortem. LSAOC specific survival analysis indicates overall survival around 80% at 2 years. This is shown in Fig. 3. Advanced stage disease was associated with poorer prognosis (Fig. 4). This did not reach significance (*p* = 0.11 student *t*-test), however there were very few women in the advanced disease cohort.

All MMR mutated women were offered gynecological malignancy surveillance from 1998. The mean duration of surveillance was 50 months. Of our cohort of LSAOC, five women had undergone regular annual surveillance for gynecological malignancies. Two LSAOC were detected through the gynecological cancer surveillance program. Both were found on transvaginal ultrasound and subsequently identified as FIGO 2009 1c endometrioid ovarian cancer and 1c clear cell ovarian carcinoma aged 33 and 34 years respectively; both remain alive without recurrence 12 and 11 years post diagnosis. Three other women presented with incidental LSAOC at the time of surgery for screen-detected stage 1a endometrial cancer (*n* = 2) or atypical endometrial hyperplasia (*n* = 1). Two of these women had occult, microscopic stage 1a or 1b OC; the third had stage 2 disease. The latter patient's ovarian tumor was not visualized on ultrasound scan during uterine surveillance, but she had an elevated serum CA125 level. All five women with LSAOC who had been under gynecological surveillance are alive and well. The majority of other LSAOC women were diagnosed with Lynch syndrome after their OC diagnosis (*n* = 41).

## 4 Discussion

Here we present the largest single institution cohort study of ovarian cancer in proven Lynch syndrome carriers and the first from the United Kingdom. We add to the body of evidence that LSAOC presents at an earlier age than OC in non-Lynch syndrome carriers. The lifetime cumulative risk of OC in our cohort was 20%. It is likely that this is an overestimate as we have not corrected for testing bias, whereby affected family members are more likely to be tested for LS. In order to adjust for this, analyses need to take into account untested first-degree relatives who may still have up to a 50% chance of carrying the mutation. Our previous work accounting for this estimated cumulative lifetime risk of OC to be closer to 6–8% [10]. Here, we show that LSAOC generally presents at an early stage, in keeping with previous reports [10], [11]. The most common histological subtype in our cohort was endometrioid adenocarcinoma but high-grade serous tumors were also seen.

Previous work suggests that the lifetime risk of OC in Lynch syndrome is around 6–14% [12] depending on the particular gene that is mutated. A 20% lifetime risk of OC for *MLH1,* 24% *MSH2* and a 1% risk for *MSH6* mutation was reported in one large series of carriers from 537 families [4]. In our cohort, similar proportions of women with *MLH1*, *MSH2* and *MSH6* mutations developed OC (Fig. 1), however only one woman with a *PMS2* mutation developed OC. She had a bi-allelic *PMS2* mutation and thus a diagnosis of constitutional mismatch repair deficiency (CMMRD) rather than Lynch syndrome. There were no cases of OC amongst our 21 heterozygous *PMS2* mutation carriers.

This study adds to the substantial evidence reporting an earlier age of onset of LSAOC compared to sporadic OC [10], [13], [14], [15]. The median age was 48 years and 79% of women in our cohort were under the age of 50 when they were diagnosed with OC. This compares to a median age of 63 years in the general population [16]. Our data highlight the importance of a low threshold for Lynch syndrome diagnostic testing alongside BRCA testing in women who present with OC under the age of 50 years. Established clinical criteria for Lynch testing, specifically the Bethesda guidelines, are not sensitive in the diagnosis of LSAOC, with only 9 women meeting the criteria.

Historically, ovarian cancer has been categorized based on morphology into Type I and Type II disease [16], although modern genetic approaches call into question the utility of such an approach, favoring genetic categorization based on mutation status as it better predicts prognosis and treatment response [16], [17], [18]. Indeed there is evidence that LSAOC is genetically distinct from sporadic OC [19]. Nonetheless, Type I disease is typically low grade with an improved overall survival rate compared with high grade, type II disease [16], [18]. We found a predominance of Type I tumors in our cohort, with over 50% of endometrioid morphology. This may help to explain the good survival rates in our population. In non-Lynch syndrome and BRCA-associated OC, high-grade serous cancers predominate [17]. We found a smaller proportion of high-grade serous OC in our cohort, similar to the results of Helder-Woolderink et al. [9]. Their systematic review draws its histological projections from studies where subjects were not proven Lynch syndrome carriers, in contrast to the current study [15]. The 10-year overall survival of LSAOC was 75% in our cohort. This compares with a 10-year survival of 35% for non-Lynch syndrome associated OC [19]. Most of the LSAOC in our cohort (65%) presented at FIGO stage 1 with relatively few (17%) OC-related deaths. Deaths attributed to OC occurred in women with endometrioid, mixed or clear cell tumors, but not high grade serous tumors, all but two of whom (*n* = 4, 66%) had stage 1 disease at presentation.

Strengths of our study include the large number of individuals in our dataset (*n* = 1047), all with documented germline mutations in one of their MMR genes. A dedicated data manager prospectively maintains our database to ensure its accuracy and completeness. It forms the basis by which clinical follow-up is organized and is regularly audited for quality assurance. Cause of death is confirmed through vertical sources including the National Cancer Registry and through death certification. This ensures the robustness of our survival analyses. Our study is limited by small numbers because LSAOC is rare. The accuracy of the cause of death data is uncertain since it is based on expert opinion in death certification rather than post-mortem findings. We also have limited events for our survival analysis; this is especially true of deaths in advanced staged disease, mainly because most women presented early. Our work needs validation through international collaboration. Our survival data are comparable with the largest cohort described in the literature [10]. However, only prospective studies can fully investigate the impact of stage, early detection and treatment modality on survival from LSAOC.

OC prevention and early detection may improve disease specific outcomes in the general population and BRCA mutation carriers [17], [18], [19]. The evidence for such an impact in LSAOC is poorly established. There is general consensus that women with Lynch syndrome should be offered risk-reducing prophylactic hysterectomy and bilateral salpingo-oophorectomy at around 45 years of age [20], [21], [22]. The utility of OC surveillance in Lynch syndrome is not yet evidence based [23]. Nonetheless, we are encouraged by the results in our cohort with two of five OC's detected at stage 1 through our local surveillance program, and another three with occult disease diagnosed at hysterectomy for screen-detected endometrial abnormalities. Surveillance can be tailored to individual women since *MSH6* carriers have a high risk of endometrial cancer but a lower risk of OC [14], [24]. Women with *PMS2* mutations appear to be at lowest risk of OC. Large collaborative retrospective studies, or adequately powered prospective studies, are needed to provide new insights into LSAOC.

## Contribution to authorship

All authors contributed to study design, data collection and interpretation. NR and EC prepared the first draft of the manuscript. All authors reviewed and agreed the final version of the manuscript.

## Conflicts of interest

The authors report no conflicts of interest.

## Funding

NR is an MRC Doctoral Research Fellow (MR/M018431/1). EJC is a National Institute for Health Research (NIHR) Clinician Scientist (NIHR-CS-012-009) and DGE an NIHR Senior Investigator (NF-SI-0513-10076). This article presents independent research funded by the National Institute for Health Research (NIHR) and facilitated by the Greater Manchester Local Clinical Research Network. The views expressed are those of the authors and not necessarily those of the NHS, the NIHR or the Department of Health.

## Figures and Tables

**Table 1:** Tabulated clinical information regarding LSAOC and subsequent cancer diagnoses. Only cases with near complete data sets are shown. *Abbreviations*: NK: not known, CRC: colorectal cancer, AH: atypical hyperplasia, AC: adjuvant chemotherapy, EC: endometrial cancer, TCC: transitional cell carcinoma, RCC, renal cell carcinoma, DCIS: ductal carcinoma in situ. *signifies woman whose AH or EC was detected during gynecological surveillance and who was found to have a synchronous OC following hysterectomy and removal of both ovaries; ** signifies concurrent cancer diagnosis at time of LSAOC diagnosis. #signifies cause of death was attributed to OC. § is a patient with bi-allelic PMS2 mutation and therefore constitutional mismatch repair deficiency (CMMRD) rather than LS and is included here for information only.

ID	Mutation	Tumor	Age at diagnosis	Year of diagnosis	Screen detected	FIGO (2009)	Treatment	Outcome	Other Neoplasms
1	*MLH1*	Endometriod	42	2006	No	1b	Surgery	Alive	Nil
2	*MLH1*	Mixed	52	1997	No	2a	Surgery	Dead (1997)	Nil
3	*MLH1*	Clear cell	47	2004	No	1a	Surgery	Dead # (2006)	Nil
4	*MLH1*	Endometrioid	38	2012	No*	2c	Surgery	Alive	Dukes A CRC and AH**
5	*MLH1*	Endometrioid	37	1997	No	1b	Surgery + AC	Alive	Nil
6	*MLH1*	Clear cell	60	2005	No	2b	Surgery + AC	Alive	Nil
7	*MLH1*	Endometrioid	45	1960	No	1a	Surgery	Dead (1996)	EC**
8	*MLH1*	Endometrioid	46	1988	No	3a	Surgery	Dead # (1989)	EC**
9	*MSH2*	Endometrioid	24	1956	No	1a	Surgery	Alive	Breast (1991 &2006) Ureteric (1986) Skin (numerous) EC (1965)
10	*MSH2*	High grade serous	47	2003	No	1c	Surgery	Alive	EC**
11	*MSH2*	Mixed	33	2011	No	3c	Surgery + AC	Alive	Nil
12	*MSH2*	High grade serous	34	1973	No	2a	NK	Dead (1991)	Nil
13	*MSH2*	Endometrioid	41	2008	No*	1a	Surgery (?AC)	Alive	EC** Brenner tumor (benign) left ovary
14	*MSH2*	Other	48	2005	No	1a	Surgery + AC	Alive	Nil
15	*MSH2*	Clear cell	34	2005	Yes	1c	Surgery	Alive	Caecum Dukes CRC 32 Sigmoid Dukes CRC 34
16	*MSH2*	Endometrioid	51	2014	No	1a	Surgery + AC	Alive	Nil
17	*MSH2*	High grade serous	25	1988	No	1c	Surgery	Alive	Nil
18	*MSH2*	Endometrioid	42	1999	No	1a	Surgery	Dead# (2000)	Nil
19	*MSH2*	High grade serous	50	1999	No	1c	Surgery	Alive	Nil
20	*MSH2*	Endometrioid	41	2009	No	1c	Surgery	Alive	Nil
21	*MSH2*	Endometrioid	41	2012	No	1b	Surgery	Alive	Nil
22	*MSH2*	Carcinosarcoma	41	1994	No	2a	Surgery	Dead (1997)	EC**
23	*MSH2*	Endometrioid	51	2005	No	1c	Surgery	Dead# (2006)	Nil
24	*MSH2*	High grade serous	70	2005	No	3b	Surgery + AC	Alive	Dukes C CRC (1982) TCC bladder (2004) RCC (2007)
25	*MSH2*	Endometrioid	43	1997	No	1a	Surgery	Alive	DCIS (1989)
26	*MSH2*	Endometrioid	57	2011	No	2a	NK	Alive	EC**
27	*MSH2*	High grade serous	67	2006	No	1a	Surgery	Alive	TCC (1997)
28	*MSH2*	Endometrioid	41	2008	No*	1b	Surgery	Alive	EC**
29	*MSH2*	Clear cel	46	1984	No	NK	Surgery	Dead# (1985)	Oesophageal (1969)
30	*MSH2*	Endometrioid	55	1986	No	3a	Surgery	Dead (2011)	Duke C CRC (1989)
31	*MSH2*	Endometrioid	33	2004	Yes	1c	Surgery	Alive	Nil
32	*MSH6*	Mixed	67	2012	No	4	Surgery + AC	Dead# (2015)	Dukes B CRC (2012)
33	*MSH6*	Other	33	1971	No	1a	missing data	missing data	missing data
34	*MSH6*	Endometrioid	46	1992	No	1a	Surgery	Alive	Nil
35	*MSH6*	Mixed	49	2010	No	2a	Surgery + AC	Alive	Nil
36	*MSH6*	Endometrioid	44	2001	No	1b	Surgery + AC	Alive	Benign dermoid cyst right ovary
37 §	*PMS2* (homozygote)	Mixed	27	2012	No	1c (both)	Surgery	Alive	Gastric Adenocarcinoma EC**

**Table 2:** Distribution of histopathology by Lynch syndrome gene mutation.

Histological type	Number	Mutation type	Number
Endometrioid	19	*MLH1*	5
		*MSH2*	12
		*MSH6*	2
Clear cell	4	*MLH1*	2
		*MSH2*	2
		*MSH6*	0
High grade serous	6	*MLH1*	0
		*MSH2*	6
		*MSH6*	0
Carcinosarcoma	1	*MLH1*	0
		*MSH2*	1
		*MSH6*	0
Mixed	4	*MLH1*	1
		*MSH2*	1
		*MSH6*	2
Other^a^	2	*MLH1*	0
		*MSH2*	1
		*MSH6*	1

a In the other category 1 × Yolk sac (*MSH6*) and 1 × Anaplastic neuroendocrine (*MSH2*).

## References

[1] Lynch H.T., Shaw M.W., Magnuson C.W., Larsen A.L., Krush A.J. (1966). Hereditary factors in cancer: study of two large Midwestern kindreds. Arch. Intern. Med..

[2] de la Chapelle A. (2005). The incidence of lynch syndrome. Familial Cancer.

[3] Hampel H., de la Chapelle A. (2011). The search for unaffected individuals with Lynch syndrome: do the ends justify the means?. Cancer Prev. Res. (Phila.).

[4] Bonadona V. (2011). Cancer risks associated with germline mutations in MLH1, MSH2, and MSH6 genes in Lynch syndrome. JAMA.

[5] Watson P. (2008). The risk of extra-colonic, extra-endometrial cancer in the Lynch syndrome. Int. J. Cancer.

[6] Malander S. (2006). The contribution of the hereditary nonpolyposis colorectal cancer syndrome to the development of ovarian cancer. Gynecol. Oncol..

[7] Walsh M.D. (2008). Molecular, pathologic, and clinical features of early-onset endometrial cancer: identifying presumptive Lynch syndrome patients. Clin. Cancer Res..

[8] Meyer L.A., Broaddus R.R., Lu K.H. (2009). Endometrial cancer and lynch syndrome: clinical and pathologic considerations. Cancer Control: Journal of the Moffitt Cancer Center.

[9] Bewtra C., Watson P., Conway T., Read-Hippee C., Lynch H.T. (1992). Hereditary ovarian cancer: a clinicopathological study. Int. J. Gynecol. Pathol..

[10] Grindedal E.M. (2010). Survival in women with MMR mutations and ovarian cancer: a multicentre study in Lynch syndrome kindreds. J. Med. Genet..

[11] Helder-Woolderink J.M. (2016). Ovarian cancer in Lynch syndrome; a systematic review. Eur. J. Cancer.

[12] Barrow E., Hill J., Evans D.G. (2013). Cancer risk in Lynch Syndrome. Familial Cancer.

[13] Barrow E. (2009). Cumulative lifetime incidence of extracolonic cancers in Lynch syndrome: a report of 121 families with proven mutations. Clin. Genet..

[14] Moller P. (2015). Cancer incidence and survival in Lynch syndrome patients receiving colonoscopic and gynaecological surveillance: first report from the prospective Lynch syndrome database. Gut.

[15] Watson P. (2001). The clinical features of ovarian cancer in hereditary nonpolyposis colorectal cancer. Gynecol. Oncol..

[16] Holschneider C.H., Berek J.S. (2000). Ovarian cancer: epidemiology, biology, and prognostic factors. Semin. Surg. Oncol..

[17] Lu K.H. (2008). Hereditary gynecologic cancers: differential diagnosis, surveillance, management and surgical prophylaxis. Familial Cancer.

[18] Evans D.G. (2009). Screening for familial ovarian cancer: poor survival of BRCA1/2 related cancers. J. Med. Genet..

[19] Jayson G.C., Kohn E.C., Kitchener H.C., Ledermann J.A. (2014). Ovarian cancer. Lancet.

[20] Schmeler K.M. (2006). Prophylactic surgery to reduce the risk of gynecologic cancers in the Lynch syndrome. N. Engl. J. Med..

[21] Koornstra J.J. (2009). Management of extracolonic tumours in patients with Lynch syndrome. Lancet Oncol..

[22] Lindor N.M. (2006). Recommendations for the care of individuals with an inherited predisposition to Lynch syndrome: a systematic review. JAMA.

[23] Auranen A., Joutsiniemi T. (2001). A systematic review of gynecological cancer surveillance in women belonging to hereditary nonpolyposis colorectal cancer (Lynch syndrome) families. Acta Obstet. Gynecol. Scand..

[24] Bonadona V. (2011). Cancer risks associated with germline mutations in MLH1, MSH2, and MSH6 genes in Lynch syndrome. JAMA.

